# MAPKAP Kinase-2 Drives Expression of Angiogenic Factors by Tumor-Associated Macrophages in a Model of Inflammation-Induced Colon Cancer

**DOI:** 10.3389/fimmu.2020.607891

**Published:** 2021-02-23

**Authors:** Lucia Suarez-Lopez, Yi Wen Kong, Ganapathy Sriram, Jesse C. Patterson, Samantha Rosenberg, Sandra Morandell, Kevin M. Haigis, Michael B. Yaffe

**Affiliations:** ^1^ Center for Precision Cancer Medicine, Koch Institute for Integrated Cancer Research and Departments of Biological Engineering and Biology, Massachusetts Institute of Technology, Cambridge, MA, United States; ^2^ Department of Cancer Biology, Dana Farber Cancer Institute, Boston, MA, United States; ^3^ Divisions of Acute Care Surgery, Trauma and Surgical Critical Care, and Surgical Oncology, Department of Surgery, Beth Israel Deaconess Medical Center, Boston, MA, United States

**Keywords:** signal transduction, MAPKAP kinase 2, tumor associated macrophage (TAM), inflammation driven tumorigenesis, colon tumors, stress signaling, angiogenesis

## Abstract

Chronic inflammation increases the risk for colorectal cancer through a variety of mechanisms involving the tumor microenvironment. MAPK-activated protein kinase 2 (MK2), a major effector of the p38 MAPK stress and DNA damage response signaling pathway, and a critical regulator of pro-inflammatory cytokine production, has been identified as a key contributor to colon tumorigenesis under conditions of chronic inflammation. We have previously described how genetic inactivation of MK2 in an inflammatory model of colon cancer results in delayed tumor progression, decreased tumor angiogenesis, and impaired macrophage differentiation into a pro-tumorigenic M2-like state. The molecular mechanism responsible for the impaired angiogenesis and tumor progression, however, has remained contentious and poorly defined. Here, using RNA expression analysis, assays of angiogenesis factors, genetic models, *in vivo* macrophage depletion and reconstitution of macrophage MK2 function using adoptive cell transfer, we demonstrate that MK2 activity in macrophages is necessary and sufficient for tumor angiogenesis during inflammation-induced cancer progression. We identify a critical and previously unappreciated role for MK2-dependent regulation of the well-known pro-angiogenesis factor CXCL-12/SDF-1 secreted by tumor associated-macrophages, in addition to MK2-dependent regulation of Serpin-E1/PAI-1 by several cell types within the tumor microenvironment.

## Introduction

Colorectal cancer is the third most common cancer type, and the second leading cause of cancer-related deaths worldwide (1). Chronic inflammation is an important contributor to tumor development and progression in various types of cancer, including those arising in the GI tract. Patients with inflammatory bowel disease (IBD), for example, are at high risk of developing colon tumors, particularly those with ulcerative colitis (UC) (2). Many of these inflammatory effects are mediated through the tumor microenvironment, including the recruitment of various types of innate and adaptive immune cells, as well as modulation of local stromal and endothelial cells as the tumors evolve.

Macrophages constitute one of the most frequent tumor-infiltrating cell types. Once tumors are established, tumor-associated macrophages (TAMs) promote tumor growth and are required for angiogenesis, invasion, and metastasis (3). Not surprisingly, high TAM content generally correlates with poor prognosis (4). In broad terms, macrophages have been generally classified into M1 and M2 types (5). M1 macrophages are activated by IFN-γ and microbial products, express high levels of proinflammatory cytokines (TNF-α, IL-1, IL-6, IL-12 or IL-23), major histocompatibility complex (MHC) molecules, and nitric oxide synthase. These M1 macrophages are capable of killing pathogens and can prime anti-tumor immune responses. By contrast, M2 or ‘‘alternatively’’ activated macrophages, are induced *in vitro* by IL-4 and/or IL-13, downregulate MHC class II and IL-12 expression, and show increased expression of the anti-inflammatory cytokine IL-10, scavenger receptor A, and arginase. TAMs often express many genes typical of the M2 phenotype and have therefore been described as ‘M2-skewed’. However, there is evidence that suggests that the phenotype of TAMs vary with the stage of tumor development. M1-like cells often predominate at sites of chronic inflammation where tumors can develop, and then are replaced by M2-like macrophages as the tumor begins to invade, vascularize and develop (6). Tumor growth and metastasis requires an increased intratumoral blood supply, which is normally triggered by tumor hypoxia. Infiltrated TAMs respond to hypoxic signals by producing pro-angiogenic cytokines and growth factors such as angiopoietin 2, vascular endothelial growth factor (VEGF), IL-8, CXCL1, and FGF-2, among others, to induce the recruitment, proliferation and maturation of endothelial cells to create new blood vessels, in a process referred to as “the angiogenic switch” (7). The signaling pathways responsible for changing the phenotypes of tumor-associated macrophages during the process of tumorigenesis and progression, including the angiogenic switch, remain incompletely defined (8, 9).

One commonly accepted model of tumor development posits that cancers co-opt many of the normal wound repair signals that non-cancerous tissues use to respond to injury, stress, and DNA damage (10, 11), in order to grow, remodel their surrounding stroma, become vascularized, and create an immune suppressive microenvironment. Our long-standing interest in stress, cell injury, and protein kinase-mediated DNA damage signaling, both within epithelial tumor cells, and between the supporting stromal and immune cells and the developing tumor, led us to study specific signaling pathways in inflammation-induced tumor progression, and in the response of growing tumors to cytotoxic treatments, using systems biology approaches (12–15). One particularly important pathway that emerged from these studies is the p38MAPK pathway, and its downstream effector kinase MAPKAP Kinase-2 (MK2). This pathway is critical for production of inflammatory cytokines and chemokines in macrophages, including TAMs (16, 17). In response to inflammatory stimuli (i.e. Toll-like receptors, TNF-α or IL-1 receptor activation for example), MAPK kinase-3 and 6 (MKK3 and MKK6) phosphorylate and activate p38α (18), which, in turn, phosphorylates and activates MK2, with which it forms a stable heterodimeric complex in the nucleus. p38α phosphorylation of MK2 exposes its nuclear export signal (NES), resulting in export of both active p38α and active MK2 out of the nucleus (19, 20). In the cytoplasm, MK2 phosphorylates Tristetraprolin (TTP), an RNA-binding protein that normally binds to AU-rich elements (ARE) in cytokine-encoding mRNAs, targeting them for degradation in the absence of inflammatory stimuli. Phosphorylation of TTP by MK2 mediates the formation of a TTP: 14-3-3 protein complex, leading to TTP release from AREs and increased cytokine mRNA translation (21, 22). Pro-inflammatory cytokines like IL-1β, IL-4, IL-6, IL-8, GM-CSF, IFN-γ, TNF-α and COX2 are post-transcriptionally up-regulated by MK2 by this mechanism (23). In addition, a novel alternative role for the p38α-MK2 pathway in suppressing cytokine production through TTP in an mRNA stability-independent manner has recently been reported in TAMs under conditions of constitutive inflammation within the tumor microenvironment, likely limiting the extent of total inflammatory cytokine production within the tumors (24).

We recently reported that genetic depletion of MK2 within the myeloid compartment—neutrophils, dendritic cells, macrophages, etc.—reduced inflammatory colon tumor progression and impaired tumor neo-angiogenesis. In addition, we showed that MK2 was required for *in vitro* macrophage polarization into the pro-angiogenic M2 phenotype (25). Here we further explore the specific importance of macrophages and MK2 function *in vivo* during inflammation-induced tumor development, using RNA-Seq, direct assays of angiogenesis factors and macrophage reconstitution experiments. We describe how MK2-depleted macrophages show reduced expression of pro-angiogenesis factors, both *in vitro* and *in vivo*, and how reconstitution of MK2 function in the macrophage compartment is both necessary and sufficient to restore angiogenesis factor expression, efficient angiogenesis and tumor progression.

## Material and Methods

### Mice

MK2 KO mice were generated as described previously (25) and maintained in a C57BL/6N background. In order to deplete MK2 in myeloid linages (LysM-KO mice), mice carrying a floxed allele of MK2 (MK2 FL/FL) were crossed with Lyz2 tm1(cre)Ifo strain (26). LysM-KO and non Cre-carrier littermate controls (LysM-FLFL) were backcrossed 5 generations to C57BL/6N.

### Murine Bone Marrow-Derived Macrophage (BMDM) Differentiation and Polarization

Bone marrow cells were isolated from femurs and tibias of MK2 WT and MK2 KO mice by briefly centrifuging the bones at 15,000 x g for 15s at 4°C in a 1.5 ml eppendorf tube. After red blood cell lysis in RBC lysis buffer (eBioscience), 2x10^6^ cells per ml were seeded on 10cm bacterial plates in IMDM supplemented with 10ng/ml murine M-CSF. An equal volume of M-CSF supplemented media was added on top of the existing media 72 h post-seeding. On day 7 after seeding, cells were detached using 5 mM EDTA in PBS w/o Ca and Mg, and re-plated on 6 cm tissue culture plates at 4 million cells per plate in IMDM (10% FBS with antibiotics). Cells were allowed to attach for 12 h. To induce polarization, cells were either unstimulated, or treated with 10 ng/ml murine IL-4 (Gibco to obtain M0 and M2 cells respectively and harvested 24h post-stimulation.

### RNA Extraction and RNA Sequencing

RNA from macrophage cultures was obtained using TRIzol (Invitrogen) and further purified using the RNeasy Mini Kit (Qiagen), following manufacturer’s instructions. RNA quality was measured using the Bioanalyzer (Agilent), to ensure RQN values were above 9 before submitting the samples for RNAseq. 50ng RNA was submitted per sample to the MIT BioMicro Center for Illumina library preparation and sequencing. Briefly, libraries were prepared for sequencing using the Kapa Hyperprep kit (Roche) and fragments were verified to be around 200bp. 40nt single-end sequencing was performed on HiSeq2000. The quality of the RNA-seq data was assessed using the FastQC [v0.11.7 (27),] tool prior to downstream analysis.

### Gene Mapping and Gene Annotation

Single-end RNA-Seq data was mapped to the *Mus musculus* genome (GRCm38 (mm10) build from Gencode (28), using RSEM (RNA-Seq by Expectation maximization) with Bowtie 2 as the aligner (29). Expected counts, expected counts rounded, TPM and FPKM files were generated.

### EdgeR

EdgeR was used to perform differential gene expression analysis using the expected counts rounded values. Exact test was performed for each pairwise comparison (MK2-WT M0 vs MK2-WT M2 macrophages, MK2-WT M0 vs MK2-KO M0 macrophages, MK2-WT M0 vs MK2-KO M2 macrophages, MK2-WT M2 vs MK2-KO M0 macrophages, MK2-WT M2 vs MK2-KO M2 macrophages, MK2-KO M0 vs MK2-KO M2 macrophages) (30, 31). Resulting data from the edgeR analysis between M2 macrophages isolated from MK2 WT and MK2 KO mice was visualized using EnhancedVolcano (32).

### GeTMM

To allow for both inter- and intra-sample comparison, we used the normalization algorithm GeTMM (Gene length corrected TMM), which combines gene-length correction (required for intrasample comparison) with the normalization procedure TMM (Trimmed Mean of M-values; required for intersample comparison) (33). Z-scores were calculated for GeTMM normalized data (FDR<0.05) and visualized as a heatmap using the software MeV v4.8.1. Pearson correlation based hierarchical clustering was performed and the sample tree/edge lengths were plotted in Dendroscope (v3.5.10).

### GSEA

Hypergeometric GSEA was performed on DE genes from edgeR analysis between M2 macrophages isolated from MK2 WT and MK2 KO mice (FDR<0.05, fold change -/+ 2) and overlaps with GO biological processes (C5, BP) were computed. Following hierarchical unsupervised clustering, hypergeometric GSEA was also performed on the DE genes from cluster 6 and overlaps with GO biological processes (C5, BP) were computed (34–36).

### Protein Array

Angiogenesis-related proteins were analyzed using the Mouse Angiogenesis Proteome Profiler™ Array Kit (R&D Systems), following manufacturer’s instructions. This immunoassay allows the simultaneous analysis of 31 angiogenesis-related proteins, shown in **Supplemental Table 1**. Bone-Marrow derived macrophages were obtained from MK2 WT and KO mice as described above, plated at the same cellular density (1X10^6^ cells/ml in a total 3 ml of culture) and treated with IL-4 to induce M2 polarization. Twenty-four hours after M2 induction, the same volume of cell culture supernatants (500 µl) was collected and diluted 1:3 for protein array assay. Membranes were scanned and pixel intensity of each spot was quantified in ImageJ, after background subtraction.

### Murine Colitis-Associated Cancer

Inflammation-induced colitis associated cancers were generated in WT and MK2 KO mice as described previously (25). In brief, 8–12 weeks old male mice were administered 2.5% DSS (MP biochemicals) in the drinking water for 5 days every 21 days, for a total of 5 cycles to induce chronic inflammation. Colon tumors were generated by intraperitoneal administration of 10 mg/ml of Azoxymethane (AOM, Sigma) before chronic DSS administration (37). Colons were harvested and tumors examined 100 days after AOM administration under a dissecting scope. Tumor images were taken and tumor size was measured using ImageJ (38).

All mouse studies were approved by the MIT Institutional Committee for Animal Care and conducted in compliance with the Animal Welfare Act regulations and other federal statutes relating to animals and experiments involving animals and adhere to the principles set forth in the Guide for the Care and Use of Laboratory Animals, National Research Council, 1996 (Institutional Animal Welfare Assurance No. A-3125–01).

### Macrophage Depletion

To deplete macrophages during AOM/DSS induced colon tumorigenesis, mice were intraperitoneally administered 1mg of anti-CFSR1 antibody (BioXcell) once weekly in the AOM/DSS protocol starting right before the fourth cycle of DSS (day 68) until the end of the protocol (day 100), as previously described (39). Control animals were administered Rat IgG2A as an isotype control as recommended by the manufacturer.

Macrophage depletion efficiency was determined by FACS mediated-quantification of CD45+CD11b+F4/80+ cells, both in the colonic mucosa and peritoneal cavity, of mice administered with 1mg IgG or anti-CFSR1 7 days after one single injection of 1mg of antibody. For lamina propria macrophages, colons were flushed with cold PBS and minced with a razor blade and digested with Liberase TL (0.3mg/ml)/DNAse (10µg/ml) solution for 40 min at 37°C. Digested tissue was then filtered through 100uM mesh and washed in PBS solution containing 1% FBS, 2mM EDTA. Peritoneal resident macrophages were obtained by peritoneal lavage using 5 mL of ice- cold PBS supplemented with 2% FBS. Single cell suspensions were incubated for 10 min at room temperature with Fc blocking (CD16/32) antibody (eBioscience) prior to staining with fluorochrome-conjugated antibodies against mixtures of the following antigens: CD45, CD11b and F4/80. DAPI was used to exclude dead cells. Multiparameter analysis was performed on a LSR Fortessa (BD) and bi-dimensional dot plots were generated using FlowJo software.

### Macrophage Adoptive Transfer

Bone-marrow cells were obtained from 8–10 weeks old male C57BL/6N mice as described above. Cells were cultured in IMDM media supplemented with 15% L-929 conditioned media to maximize cell numbers. To generate conditioned media, L-929 cell line was cultured in IMDM 10% FBS with antibiotics for 7 days. Media was collected, filtered through 0.22uM filters and stored at −80°C for later use.

After seven days in culture, macrophages were detached and counted, resuspended in sterile PBS and intraperitoneally administered to mice. One million cells per mouse was administered once weekly to mice under AOM/DSS protocol, from day 68 to the end of the experiment (Day 100).

### Immunohistochemistry

Harvested colons were flushed with PBS and Swiss-rolled prior to fixation in 10% neutral buffered formalin and paraffin embedding. Four micron sections were de-waxed and rehydrated before heat-mediated antigen retrieval in citrate buffer (pH 6). Anti-Serpin-E1 (Thermo-scientific MA5-17171, 1:1,000), anti-Cxcl12 (R&D systems MAB350-SD, 1:200), anti-Timp1 (Thermo-scientific MA5-13688 1:100), Anti F4/80 (CST 70076S, 1:250), anti-Arg1 (CST 93668S, 1:100), anti-iNOS (CST 13120S, 1:400), anti-CD31 (Abcam, ab28364, 1:200) antibodies were used for immunohistochemistry. Impact DAB (Vector) secondary antibodies were used, and samples were hematoxylin counterstained before mounting. Slides were scanned in a Leica slide scanner before analysis, which was performed using Aperio ImageScope software. Leica’s “positive cell count” algorithm was used for automated counting of positive cells in all immunostainings, except CD31 and F4/80 staining, where the staining pattern does not allow easy automated identification of single cells. In these cases, the “positive pixel count” algorithm was used instead. In all cases, positive counts (cells or pixels) were normalized by the analyzed area in mm^2^.

### Statistical Methods

Unless stated otherwise, all data was plotted and analyzed in GraphPad Prism software, using Student’s t test analysis. Data represent the mean +/− SEM. For tumor size analysis, all tumor sizes from all mice within same experimental group were pooled for statistical analysis. For IHC, stained slides from serial sections of Swiss-rolled entire colons were analyzed, and all tumor areas from mice within the same experimental group were pooled for statistical analysis.

## Results

### MK2 Deficiency Markedly Alters the Transcriptional Program During M2 Macrophage Polarization

**Figure 1:**
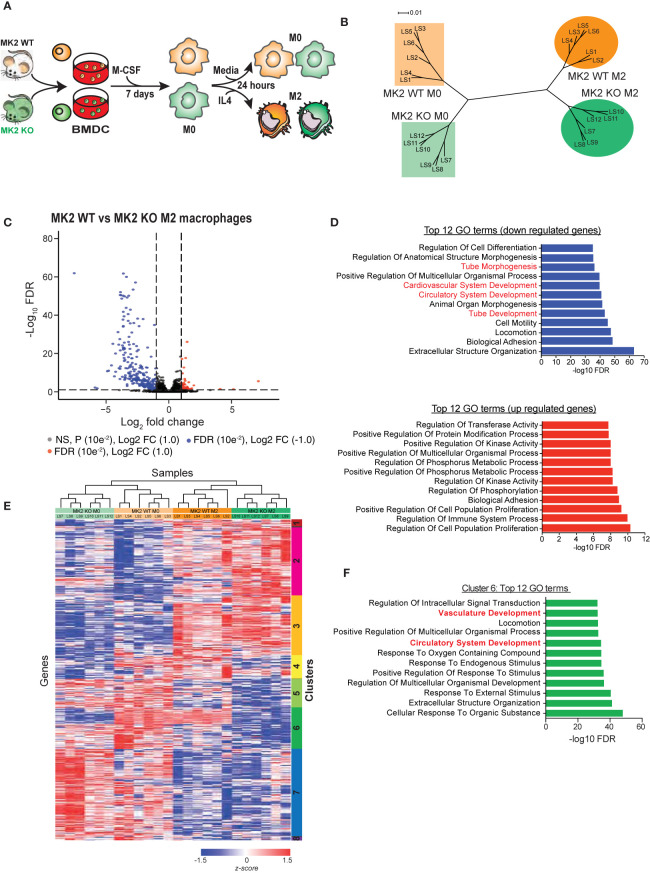
MK2 deficiency markedly alters the transcriptional program during M2 macrophage polarization. **(A)** Experimental scheme for polarization of bone marrow-derived cells from MK2 WT and MK2 KO mice into M0 and M2 macrophages. **(B)** Dendrogram visualization of unsupervised hierarchical clustering analysis of RNA-Seq data for M0 and M2-polarized macrophages derived from MK2 WT and KO mice. **(C)** Volcano plot showing the significantly expressed differential genes following edgeR analysis of M2 macrophages derived from MK2 WT and KO mice. **(D)** GSEA analysis identifies the top 12 down- and up-regulated GO terms that differ between M2-polarized macrophages derived from MK2 WT and KO mice. **(E)** Heatmap of RNA-Seq expression for genes that are differentially expressed between MK2 WT M0, MK2 WT M2, MK2 KO M0 and MK2 KO M2 (FDR <0.05, all pairwise comparisons). Expression values were converted to z-scores to facilitate visualization. **(F)** Hypergeometric test GSEA identification of the top 12 GO terms corresponding to genes in cluster 6 of panel E that are dysregulated in MK2-deficient macrophages, compared to WT macrophages, following M2 polarization.

We and others have previously described how MK2 genetic inactivation results in a significant delay in tumor progression in a mouse model of inflammation-driven tumorigenesis (25, 40–42). Interestingly, our study showed that while global numbers of macrophages were not affected by loss of MK2 function (25), alternatively polarized, M2-like macrophage recruitment was significantly reduced in the microenvironment of colon tumors. Importantly, genetic knock-out of MK2 (either constitutive or myeloid-compartment specific) resulted in a poor vascularization of tumors (25), and conditioned media from MK2-knock-out M2 macrophages exhibited reduced pro-angiogenic capacities when incubated with endothelial cells *in vitro* (25). To better understand the role of MK2 in macrophage differentiation and function, we examined the impact of knocking out MK2 on the transcriptional program that is activated during macrophage M2 polarization using RNA-Seq (**Figure 1A**). Unpolarized macrophages (M0) were isolated and propagated from the bone marrow of six MK2 wild type (WT) or from six MK2 knock out (KO) mice in the presence of the cytokine M-CSF for 7 days (43). This protocol results in highly enriched macrophages cultures (~80% purity) with similar efficiency in WT and KO cells (25). MK2 WT or MK2 KO M0 cells were then either cultured for an additional 24 h in the presence of the macrophage alternative activator IL-4 to drive M2 polarization or treated with a vehicle (PBS) control. We have previously reported IL-4 driven M2 polarization is defective in MK2 KO macrophages compared to WT [% of CD206+ cells: WT 45.35+/- 12.83 vs. KO 16.07+/-13.60 (25)]. RNA was extracted and RNAseq was then performed on these six biological replicates.

RNAseq reads were mapped to the mouse transcriptome using RSEM (RNA-Seq by Expectation Maximization) and EdgeR was used to identify differentially expressed (DE) genes during M2 macrophage polarization, and further analyzed based on MK2 status (31). Exact tests (classic EdgeR) were performed on all pairwise combinations of MK2-WT M0 macrophages, MK2-WT M2 macrophages, MK2-KO M0 macrophages, and MK2-KO M0 macrophage populations. This analysis identified a total of 4,627 differentially expressed genes with an FDR<0.05 in any of the 6 pairwise comparisons (**Supplemental Tables 2–7**). As expected, in WT MK2 macrophages, addition of IL-4 resulted in the increased expression of Arginase-1, Retnla, and MRC1 (CD206) which is indicative of M2 polarization (**Supplemental Table 1**). Furthermore, a hypergeometric test performed on all of the significantly up- and down-regulated genes (exact test; FDR <0.05 and fold-change ≥ +/− 2; 1,1017 genes) showed significant overlap with an M2 gene expression signature reported by Jablonski and colleagues (44) (up-regulated genes, p=3.61e^-14^; down-regulated genes, p=1.24e^-15^), further verifying polarization of M0 macrophages to an M2 state.

To allow for both inter- and intra-sample comparison, we used the algorithm Gene length-corrected Trimmed Mean of M-values (GeTMM), which combines gene-length correction (required for intra-sample comparison) with the normalization procedure TMM (Trimmed Mean of M-values; required for inter-sample comparison) (33). Only genes that were identified to be significantly (FDR<0.05) DE in any of the 6 EdgeR pairwise comparisons were used. We found that the six biological replicates from each group clustered tightly with each other in their groups. Interestingly, M0 macrophages from MK2 wild-type mice and M0 macrophages from MK2 knock-out mice clustered closer to each other than to their corresponding M2-polarized counterparts (**Figure 1B**), suggesting that MK2-KO M0 macrophages are more similar to MK2-WT M0 macrophages than they are to their corresponding M2-like states. Furthermore, this gene expression-based clustering indicates that genetic deficiency of MK2 in macrophages does not completely abrogate M2 polarization, since these cells cluster closer to WT M2 macrophages than to the KO M0 cells, but it significantly altered the M2 transcriptional program, since MK2-KO M2 samples clustered distinctly from the MK2-WT M2 macrophage population.

Next, we examined the genes that were dysregulated in M2-polarized macrophages upon MK2 genetic inactivation (cf. MK2-WT M2 vs. MK2-KO M2 macrophages). We identified 440 genes with FDR<0.05 with a fold change of ≥ -/+ 2 (**Figure 1C**, **Supplemental Table 4**) that were differentially expressed between WT and MK2-KO macrophages after polarization towards an M2 state. To identify the functions associated with these genes, GO biological process terms and their significance were computed using the hypergeometric distribution, and the top 12 up- and down-regulated GO terms identified (**Figure 1D**) (34–36). Interestingly, four of the top 12 down-regulated GO terms are tube morphogenesis, cardiovascular system development, circulatory system development, and tube development. These data agree with our previous study and others (25, 45, 46), that reported roles for MK2 in vasculature development and angiogenesis. Interestingly, among the 12 GO terms most enriched in genes up-regulated upon MK2 inactivation were regulation of kinase activity, phosphorus metabolism and phosphorylation, consistent with the known cellular role of MK2 as a central kinase in the p38/MAPK pathway (47).

Next, all significant DE genes identified by the EdgeR analysis which combined all 6 pairwise comparisons between groups were GeTMM normalized and used for unsupervised hierarchical clustering. As shown in **Figure 1E**, this resulted in the appearance of 8 distinct clusters of genes. Of particular interest is cluster 6, which shows the largest difference in DE genes between MK2-wild type and MK2-deficient macrophages before and after M2 polarization. Most of these genes showed striking down-regulation in the MK2 KO macrophages compared to their WT counterparts upon M2 polarization. Hypergeometric GSEA analysis of the genes in cluster 6 (**Figure 1F**) identified vasculature development and circulatory system development as particular important GO terms that showed MK2-dependent changes during M2 polarization. Taken together, these RNA-Seq data indicate that MK2 deficiency significantly alters the transcriptional program of M2 macrophage polarization, and among the genes that are mostly significantly dysregulated are those related to angiogenesis and vasculature development.

### MK2 Deficiency Halts the Production and Secretion of Pro-Angiogenic Factors by M2 Macrophages

**Figure 2:**
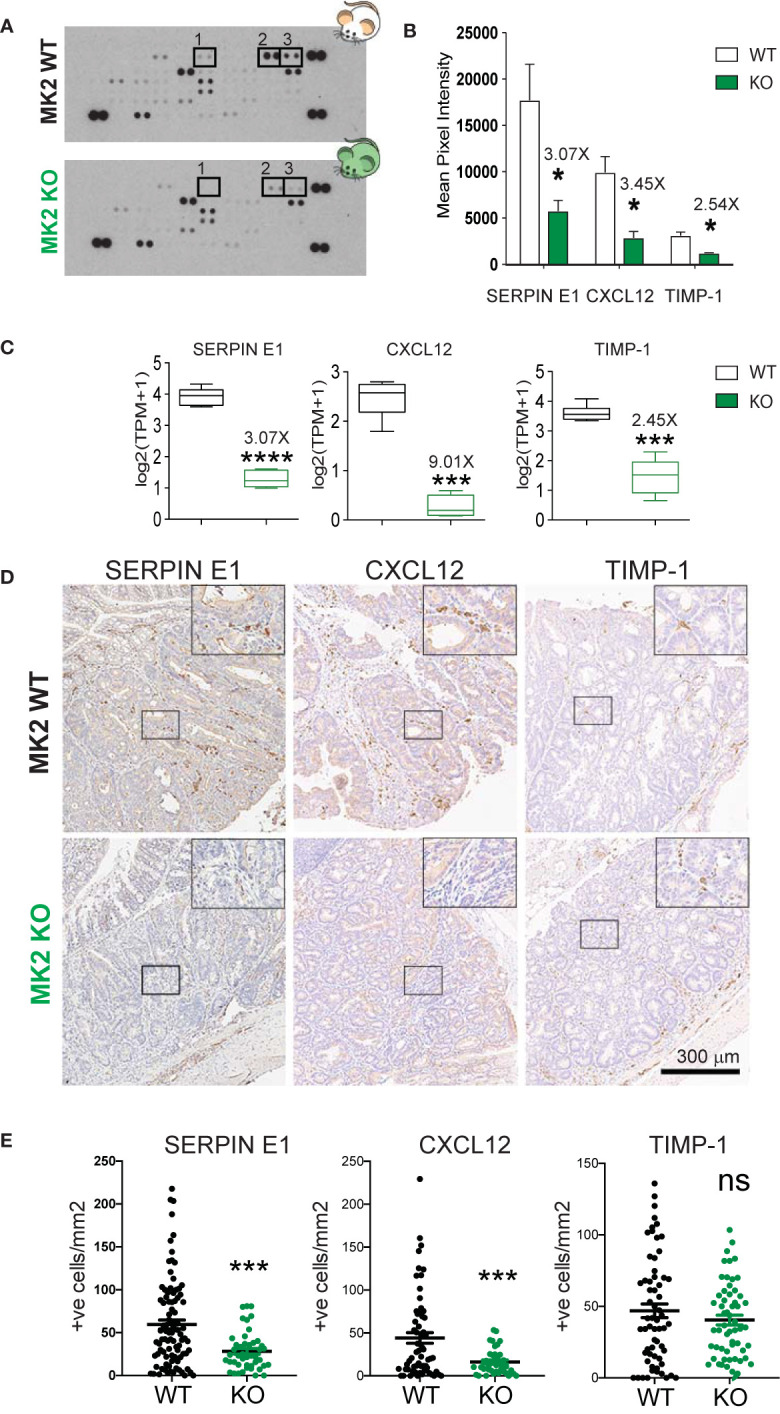
MK2 deficiency halts the production and secretion of pro-angiogenic factors. **(A)** Representative images of Mouse Angiogenesis Protein Array (R&D) membranes. Squares highlight the spots corresponding to Timp-1 (1), Serpin-E1 (2) and Cxcl-12 (3). **(B)** Protein quantification of Timp-1, Serpin-E1, and Cxcl-12 in macrophage culture supernatants from M2-polarized MK2-WT or KO macrophages. Spot intensity was quantified in ImageJ. Data corresponds to three membranes per genotype, where culture supernatants from three independent biological replicates were tested. **(C)** RNA quantification of Timp-1, Serpin-E1, and Cxcl-12 from M2-polarized MK2-WT or KO macrophages from the experiments shown **Figure 1**. Box and whisker plots display median (line), 25^th^ to 75^th^ percentiles (boxes) and min and maximum (whiskers) RNA expression levels from 6 biological replicates each of MK2-WT and KO macrophages. **(D)** Representative pictures of angiogenic factors detected by immunohistochemistry in MK2 WT (upper panel, 10 mice) and MK2 KO (lower panel, 5 mice) colon tumors. **(E)** Stained slides from serial sections of Swiss-rolled entire colons were scanned and quantified in an automated manner using the positive nuclei algorithm in ImageScope and normalized by area analyzed in mm^2^. Each data point corresponds to a single tumor area, and all tumors from all mice with the same genotype [MK2 WT (10 mice) and MK2 KO (5 mice)] are shown. Scale bar 300 µm. In panels **(B, C, E)** statistical significance was determined using the Student’s t test. *p-value < 0.05; ***p-value < 0.001, ****p-value < 0.0001; ns, not significant.

The observed transcriptomic changes indicate that MK2 is required for the expression of a large number of genes directly implicated in angiogenesis during M2 polarization. This finding is in good agreement with our previous observation that conditioned media from MK2-inhibited M2 macrophages was defective in promoting the proliferation of endothelial cells and driving their morphogenic transformation into vascular-like structures *in vitro*. To further validate these RNA expression results at the protein level, we directly measured the secretion of known angiogenic factors by M2 polarized macrophages from MK2 wild-type and KO mice. Culture supernatants from M2 polarized macrophage were harvested from both MK2 WT and KO mice and the secretion of angiogenic factors in the media was then quantified using a mouse angiogenesis antibody array (**Figure 2A** and **Supplemental Table 1**) (R&D Systems). Of the 31 angiogenesis-related factors represented on these arrays, 3 of them displayed noticeably reduced levels in the supernatants of MK2-KO M2 macrophages compared to the WT controls: Serpin-E1, Cxcl-12 and Timp-1 (**Figure 2B**). Importantly, RNA expression levels for these same factors from the transcriptome analysis shown in **Figure 1** revealed similar levels of RNA reduction in the MK2 KO M2 macrophages relative to WT controls (3.07-fold, 9.01-fold and 2.45-fold, respectively) (**Figure 2C**), as the reduction in levels of the secreted protein products (3.07-fold, 3.45-fold and 2.54-fold, respectively) (**Figure 2B**). This suggests that their loss in MK2-deficient macrophages was primarily a direct result of alterations in RNA expression rather than loss of some MK2-dependent cytoskeletal or exocytosis process.

To validate that these three angiogenesis factors are truly regulated by MK2 *in vivo*, we next measured their expression in colon tumors after MK2 depletion. Briefly, MK2 WT and KO mice were challenged with the carcinogen Azoxymethane (AOM, 10 mg/kg) followed by 5 cycles of dextran sodium sulfate (DSS, 2.5%) in the drinking water. AOM is a mutagenic agent which in combination with the inflammatory effect of DSS gives rise to visible tumors after 100 days. Following this protocol, visible adenomas develop in mice colons after 100 days (37). Of note, we have previously described that MK2 KO mice develop smaller adenomas compared to WT controls, have significantly less infiltration of M2-like macrophages and are significantly less vascularized (25). Tumors were processed for pathological examination, immuno-stained for the presence of Serpin-E1, Cxcl-12 and Timp-1 (**Figure 2D**). Positive cells within the tumors were enumerated in a blinded fashion (**Figure 2E**). As shown in **Figure 2D**, MK2 WT tumors are heavily infiltrated with cells that express Serpin-E1, Cxcl-12 and to a lesser extent, Timp-1. Interestingly, MK2 KO tumors show significantly lower infiltration of Serpin-E1 and Cxcl-12 positive cells in tumors, in good agreement with the downregulation of these factors in bone-marrow derived MK2 KO macrophages previously seen. However, the expression of Timp-1 in MK2 KO tumors was similar to MK2 WT tumors, which indicates that the regulation of this factor *in vivo* is not fully MK2-dependent. We therefore conclude that Serpin-E1 and Cxcl-12 expression, but not Timp-1 expression, is MK2-dependent, both *in vitro* and *in vivo.*

### Cxcl-12 Is Mainly Produced by Tumor-Associated Macrophages in Colon Tumors

**Figure 3:**
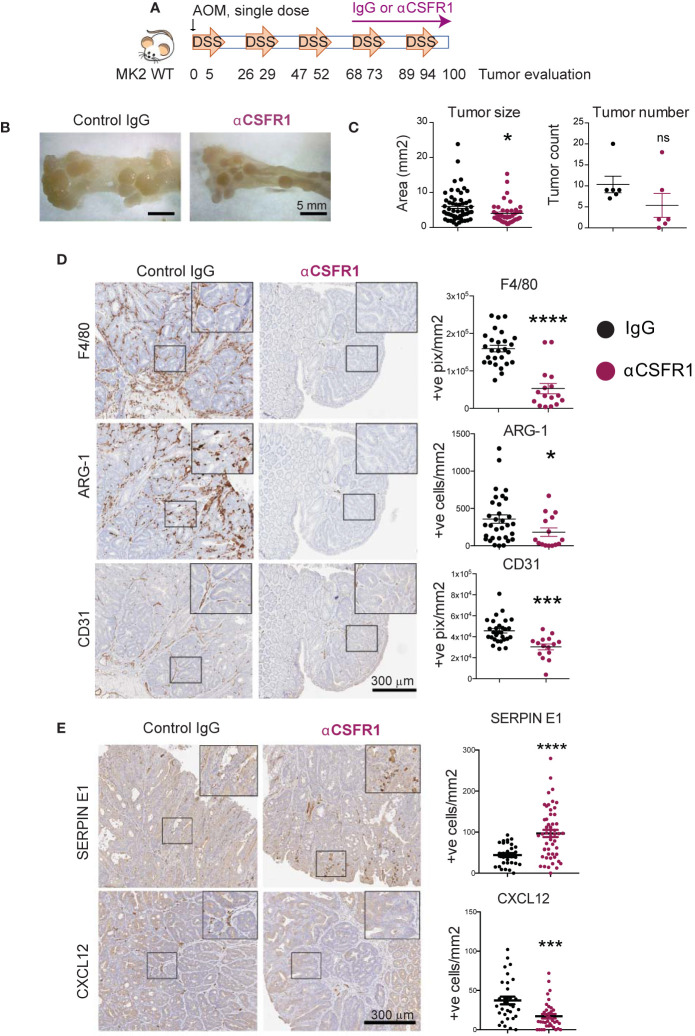
Cxcl-12 is mainly produced by tumor-associated macrophages in colon tumors. **(A)** Chronic colon inflammation was induced by 2.5% DSS administration in the drinking water for 5 days, every 21 days for a total of 5 cycles. 10 mg/kg AOM was intraperitoneally administered at the start of the treatment to generate colon tumors. Macrophages were immunodepleted with anti-CSFR1 once weekly from day 68 to the end of the protocol. IgG was used as isotype control. **(B)** Representative pictures from distal colons portions from IgG controls (left) and macrophage-depleted (right) mice at the time of tumor evaluation. Note the smaller tumor size in macrophage-depleted mice. Scale bar 5mm. **(C)** All tumors in all of the mice (6 mice total per group) were blindly counted under the dissecting microscope and tumor areas quantified using ImageJ. **(D)** Representative pictures of macrophage marker F4/80, M2 marker Arginase-1 and blood vessels marker CD31 detected by immunohistochemistry on macrophage-depleted and corresponding isotype controls. Stained slides were scanned, and positive cells were automated counted within tumor areas using the positive pixel or nuclei positive algorithm in ImageScope. Strong positive counts were normalized by area of tumor analyzed in mm^2^. Each data point reflects a single quantified tumor area from all tumors in all mice within the same treatment group. Scale bar 300 µm. **(E)** Representative pictures of angiogenic factors immunodetection on macrophage-depleted mice and corresponding isotype controls. Scanned pictures were analyzed as in **(D)** Scale bar 300µm *p-value < 0.05; ***p-value < 0.001; ****p-value < 0.0001; ns, not significant. Student’s t test.

We have shown that MK2 is required for the production of Serpin-E1 and Cxcl-12 both in *in vitro* cultured M2 macrophages and in the tumor microenvironment. To examine whether these factors are, in fact, produced by tumor-associated macrophages, we immuno-depleted macrophages in the AOM/DSS mouse model of colon cancer. Macrophage depletion in this model was accomplished using antibodies directed against CSFR1. In order to deplete macrophages during the period of tumor progression, rather than prior to tumor initiation, 1 mg of anti-CSFR1 antibody was dosed intraperitoneally weekly, starting before the fourth cycle of DSS (**Figure 3A**). Non-specific IgG was administered as an antibody control to macrophage non-depleted animals. Efficient and sustained macrophage depletion was confirmed 7 days after a single anti-CSFR1 dose in both in the peritoneal cavity and colon (**Supplemental Figure 1**).

At the end of the protocol, colons were harvested, longitudinally opened, and macroscopic tumors counted under the dissecting microscope. Pictures were taken to quantify measurements of tumor size using ImageJ. Interestingly, macrophage-depleted mice showed significantly smaller tumors than IgG treated mice, as well as a non-statistically significant trend towards fewer numbers of adenomas, as quantified in **Figures 3B, C**. Tumors were harvested, processed for pathological examination, and stained for markers of total macrophages (F4/80) and M2 alternatively-activated macrophages (Arginase-1). Blinded quantification confirmed that anti-CSFR1 efficiently depleted total macrophages in the tumor microenvironment of these mice (i.e. reduction of F4/80 positive cells, **Figure 3D**, top panels). Furthermore, anti-CSFR1 treatment also prevented the accumulation of M2-like macrophages within the tumors (**Figure 3D**, middle panels). Notably, tumor vascularization was also significantly impaired after anti-CSFR1 treatment, as assessed by staining for CD31, a marker of endothelial cells, indicating a critical role of macrophages in promoting tumor neo-angiogenesis (**Figure 3D**, lower panels). Remarkably, these *in vivo* tumor growth and angiogenesis analyses revealed that bulk depletion of macrophages during colon tumor progression phenocopied our previous results seen following genetic inactivation of MK2 in the entire myeloid compartment (25).

We next measured the expression of Serpin-E1 and Cxcl-12 in control and macrophage-depleted colon tumors, since we had shown that both of these pro-angiogenic factors are regulated by MK2 signaling in macrophages (**Figure 2E**). We confirmed that Cxcl-12 expression was significantly decreased after macrophage depletion (**Figure 3E** bottom panels), suggesting that macrophages, rather than neutrophils, are the main producers of this factor in the tumor microenvironment. However, bulk macrophage depletion did not reduce the number of Serpin-E1 positive cells in the tumor microenvironment but instead resulted in Serpin-E1 upregulation (**Figure 3E** top panels), indicating that this factor is not exclusively produced by macrophages in this context.

Taken together, these results demonstrate that macrophages are the main drivers of tumor neo-angiogenesis in inflammation-driven colon tumors, as well as the main producers of the pro-angiogenic factor Cxcl-12.

### MK2 Regulates Macrophage Production of CXCL-12 to Support Angiogenesis During Inflammation-Associated Tumorigenesis

Having shown that Cxcl-12 production in the tumor microenvironment is macrophage dependent and MK2 regulates its expression *in vitro*, we next investigated if MK2 was required for Cxcl-12 expression by macrophages *in vivo*.

**Figure 4:**
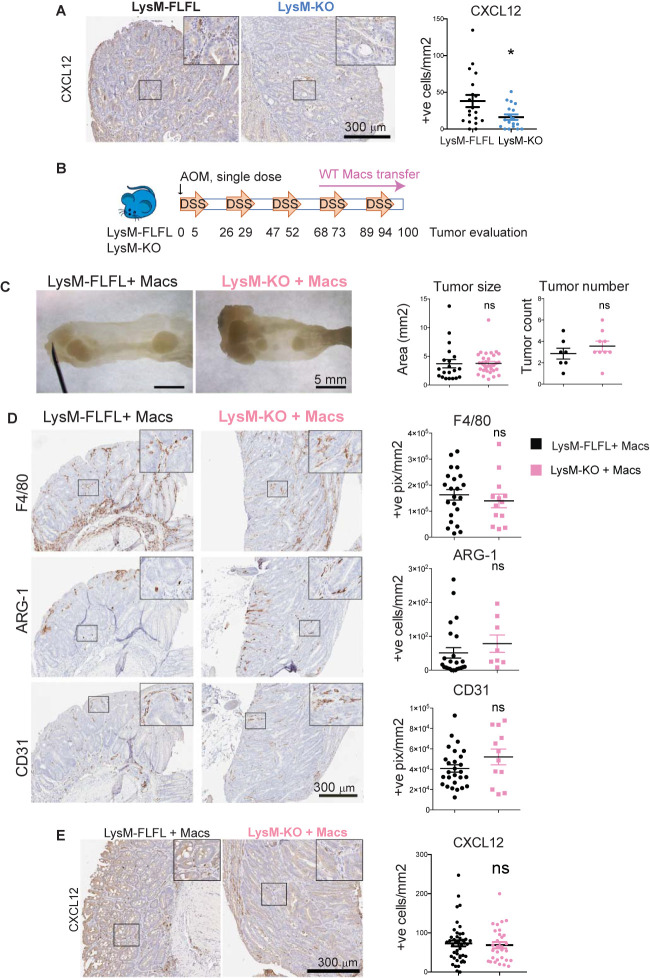
MK2 regulates macrophage production of CXCL-12 to support angiogenesis during inflammation-associated tumorigenesis. **(A)** Representative pictures of immunodetection of Cxcl-12 on myeloid-specific MK2 KO mice (LysM-KO, 7 mice) and corresponding littermate controls (LysM-FLFL, 4 mice). Stained slides from serial sections of swiss-rolled entire colons were scanned and positive cells were automated counted within tumor areas using the positive pixel algorithm in ImageScope. Strong positive pixels counts were normalized by area of tumor analyzed in mm^2^. Each data point corresponds to a single tumor area, and all tumors from all mice with the same genotype are shown. Scale bar 300μm. **(B)** Colon tumors were induced by AOM/DSS protocol and both LysM-KO and littermate controls LysM-FLFL mice were transferred with WT macrophages once weekly from day 68 to the end of the protocol. **(C)** Representative pictures from distal colons portions from LysM-FLFL (left) and myeloid-specific MK KO LysM-KO (right) mice after WT macrophage transfer. Note the similar size of tumor generated in both groups. Scale bar 5mm. Tumors from macrophage transferred LysM-FLFL (7 mice) and LysM-KO (9 mice) were blindly counted under the dissecting scope and areas were quantified using ImageJ. **(D)** Representative pictures of macrophage marker F4/80, M2 marker Arginase-1 and blood vessels marker CD31 immunodetection on colon tumors from macrophage transferred LysM-KO and LysM-FLFL mice. Stained slides from serial sections of swiss-rolled entire colons were quantified as in panel **(A)** Scale bar 300 µm. **(E)** Representative pictures of Cxcl-12 detection by immunohistochemistry on tumors from macrophage-transferred mice, and serial sections of swiss-rolled entire colons analyzed as in panels **(A, D)** Scale bar 300 µm *p-value < 0.05; ns, not significant. Student’s t test.

We first measured Cxcl-12 expression in tumor infiltrating cells in the myeloid-specific MK2 KO (LysM-MK2-KO) animals. We had previously shown that myeloid-specific depletion of MK2 results in smaller tumors in the AOM/DSS model, with less infiltration of M2 macrophages and reduced vascularization [**Supplemental Figure 2**, (25)] recapitulating the phenotype observed in the whole-body KO (25) and by macrophage depletion in this tumor model (**Figures 3B–D**). Colon tumors generated in the LysM-MK2-KO mice were surveyed for Cxcl-12 expression by immunostaining. We could confirm that Cxcl-12 expression in the tumor microenvironment of LysM-MK2-KO mice was dramatically reduced, providing direct support that myeloid MK2 is necessary for Cxcl-12 expression *in vivo* (**Figure 4A**).

Next, to demonstrate that it is specifically the macrophage MK2 activity that is sufficient to regulate Cxcl-12 expression in tumors, and therefore promote tumor progression and angiogenesis, we restored MK2 function by adoptive transfer of WT macrophages into the LysM-MK2-KO mice during AOM/DSS tumorigenesis. As proper controls, LysM-FLFL, MK2 WT littermate controls were as well adoptively transferred with WT macrophages.

MK2 proficient macrophages were derived from the bone-marrow of MK2 WT mice. After seven days in culture, one million of cells per mouse were intraperitoneally administered to both LysM-MK2-KO mice and their littermate controls. Macrophages were adoptively transferred weekly starting at the fourth cycle of DSS, as we aimed to investigate MK2 functional restoration in macrophages during tumor progression but not tumor initiation (**Figure 4B**). Colons were harvested at the end of the protocol, and tumors were counted, and their size measured under a dissecting microscope. As shown in **Figure 4C**, functional restoration of MK2 within macrophages was sufficient to restore tumor growth in the myeloid MK2 KO, since no significant differences in tumor size or number were observed between LysM-MK2-KO and their littermate controls following adoptive transfer of MK2-proficient macrophages.

We next immunostained colon tumors for the broad macrophage marker F4/80 and the M2 specific marker Arginase-1 in the WT and LysM-MK2-KO mice following reconstitution with WT macrophages. Similar number of total macrophages were observed in animals from both genotypes, but importantly, the number of Arginase-1 positive M2-like macrophages was restored to normal levels in the LysM-MK2-KO animals following reconstitution with WT macrophages (**Figure 4D**). This was in contrast to the phenotype we previously observed in LysM-MK2-KO mice, where Arginase-1 positive cells were dramatically reduced in colon tumors, despite the total numbers of macrophages being similar (**Supplemental Figure 2**). Taken together, these data indicate that reconstitution of MK2 activity within macrophages is sufficient to fully restore M2 macrophages infiltration into colon tumors *in vivo*.

We next quantified vascularization of the tumors after macrophage adoptive transfer by immunostaining for the endothelial marker CD31. As shown in the lower panel of **Figure 4D**, reconstitution of MK2 WT macrophages into the myeloid MK2 knock-out animals was sufficient to fully restore the defective angiogenesis previously observed in the LysM-MK2-KO mice [**Supplemental Figure 2** and reference (25)].

Finally, we measured the level of expression of Cxcl-12 in tumor infiltrating cells after WT macrophage transfer. As shown in **Figure 4E**, Cxcl-12 positive cells were now present in the tumor microenvironment at the same level as in the littermate MK2 WT controls (LysM-FLFL). This result indicates that MK2 is required for the effective production of Cxcl-12 by tumor-associated macrophages. Of note, infusion of WT macrophages into the MK2 WT mice resulted in increased numbers of Cxcl-12 positive cells, even higher than in not-infused controls (**Figure 4**, compare LysM-FLFL quantification in panels **A** and **E**), consistent with macrophages being the main producers of Cxcl-12 in the tumor microenvironment *in vivo*.

Taken together, our results conclusively demonstrate that it is macrophage MK2 function that is both necessary and sufficient for tumor neo-angiogenesis. Furthermore, we have demonstrated that the pro-angiogenic factor Cxcl-12 is directly regulated by MK2 signaling in macrophages *in vitro*, and its levels are markedly attenuated *in vivo* in inflammatory colon tumors that develop in mice with MK2-deficient macrophages. These findings, in combination with the reduced angiogenesis observed in myeloid MK2 knock-out animals, which is fully restored by adoptive transfer of MK2 WT macrophages into animals lacking MK2 in the myeloid compartment, strongly implicates the macrophage MK2-Cxcl-12 axis as a critical regulator of tumor neo-angiogenesis, consistent with the known role of Cxcl-12 in tumor promotion and vascularization (48, 49).

## Discussion

MK2, a key effector kinase of the p38MAPK pathway, regulates multiple critical aspects of the innate immune system cells, including neutrophils (50–52), dendritic cells (53–55) and macrophages (56, 57). There is growing evidence that p38/MK2 pathway significantly contributes to inflammation-driven tumorigenesis at multiple levels, since elegant studies from the Nebreda lab have shown that epithelial p38α activity is required for tumor maintenance while myeloid p38α regulates both inflammatory cell recruitment to tumors and chemokine production (58, 59). We have demonstrated, here, and in our previous work, that the p38MAPK downstream effector kinase MK2 is specifically required for the pro-angiogenic role of macrophages in a mouse model of inflammation-driven colon tumors. In the studies presented here, we first performed RNA expression analysis on macrophages from whole body MK2 KO mice to explore the molecular mechanisms underlying defective angiogenesis, since we had shown previously that these animals had defective colon tumor angiogenesis, and that macrophages derived from these animals *in vitro* were less efficient at polarizing into an M2 pro-angiogenic phenotype (25). Based on the RNA expression data, we showed that macrophages from these whole body MK2 KOs were defective in expression of several pro-angiogenesis factors *in vitro* (**Figures 1** and **2**). Importantly, although we had previously shown that myeloid-specific MK2 KO animals fully recapitulated the colon tumor angiogenesis-defective phenotype seen in whole body MK2 KO mice (25), it was possible that the defective tumor angiogenesis phenotype seen in the myeloid MK2 KO resulted from MK2 signaling in non-macrophage myeloid cell types such as neutrophils or dendritic cells. To eliminate this possibility, we showed here that specific depletion of the macrophage population in wild-type animals also recapitulated the whole body and myeloid MK2 KO colon tumor phenotype. Both global and myeloid-specific MK2 knock-outs, as well as bulk macrophage depletion results in similar numbers of tumors but a significant reduction in tumor size, as well as a reduction in the presence CD31+ endothelial cells within the tumors consistent with impaired tumor angiogenesis and progression [**Figure 3** and (25)]. Finally, to further prove that it is MK2 signaling within macrophages that is critical for tumor angiogenesis *in vivo*, we used adoptive transfer of WT macrophages into myeloid specific MK2 KOs. This treatment fully restored the recruitment of M2-polarized macrophages into the tumors, and reversed the defective angiogenesis phenotype (**Figures 4C, D**), indicating that the MK2 signaling/angiogenesis link is intrinsic to the macrophages, and does not arise from defective MK2 signaling in some other cellular compartment. Proangiogenic roles for MK2 have previously been described within endothelial cells, where MK2 is required for efficient post-natal arteriogenesis and vascularization in response to arterial injury or ischemia (60–62). However, our study is the first to demonstrate a specific pro-angiogenic role of MK2 within tumor-associated macrophages *in vivo*.

We observed that MK2 depleted BMDMs show deficient expression and secretion of three angiogenesis factors: TIMP-1, Serpin-E1 and CXCL-12. TIMP-1 is a secreted protein with known anti-angiogenic properties. It inhibits the action of matrix metalloproteinases (MMPs), which are involved in endothelial migration and capillary formation (63). Paradoxically, however, recent studies have shown that high levels of TIMP-1 in both plasma and tumor tissue are associated with poor prognosis in several cancers, including prostate and colon cancer (64). Although BMDMs expressed TIMP-1 in an MK2-dependent manner, we found that MK2 whole-body knock-out mice showed similar TIMP-1 levels within inflammatory colon tumors, suggesting more complex regulation of TIMP-1 in an *in vivo* setting.

Serpin-E1, also known as PAI-1, is one of the three main components of the plasminogen activation system, where it functions as the major inhibitor of tPA that limits the cleavage of plasminogen to plasmin. High levels of Serpin-E1 are associated with poor prognosis in several types of cancer and there is a large amount of *in vitro* and *in vivo* evidence of its role in favoring tumor progression and angiogenesis (65, 66). The underlying mechanism responsible for Serpin-E1 upregulation in cancer is not fully understood, but it is known that it is secreted by either epithelial, stromal or endothelial cells, where it stabilizes the extracellular matrix and favors endothelial cells migration (67, 68). In cultured endothelial cells, expression of Serpin-E1 is known to be regulated by the p38MAPK pathway (69) and the presence of Serpin-E1 in the local tumor microenvironment has been shown to promote TAM polarization towards an M2 phenotype (70), suggesting the presence of a complex feedback loop. We observed loss of PAI-1 expression in tumors from whole body MK2 KO mice, demonstrating an important role for MK2 in PAI-1 expression *in vivo*. However, PAI-1 expression was not reduced, but actually was increased in colon tumors that arose in the setting of macrophage depletion. This result suggests that non-macrophage cells types are primarily responsible for the MK2-dependent expression of PAI-1 *in vivo*, most likely in endothelial cells (69).

**Figure 5:**
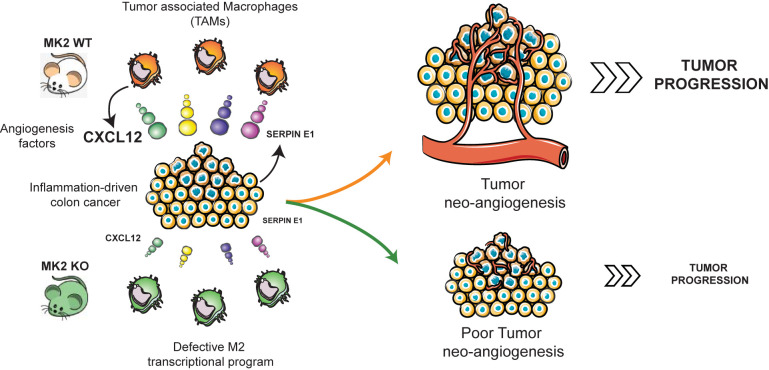
MK2 regulates the expression of Cxcl-12 in TAMs to promote angiogenesis and tumor progression. MK2 activity is required for expression of Cxcl-12 in tumor associated macrophages, and for Serpin-E1 expression within the tumor microenvironment, promoting tumor angiogenesis and progression. Mouse images courtesy of Clker/Barretto. Tumor angiogenesis, macrophages, and carcinoma images adapted from Servier Medical Art.

Cxcl-12, also known as SDF-1, is a chemokine that induces neovascularization in ischemic lesions, tumors, and wounded tissues by recruiting bone marrow stromal stem cells through its interaction with the receptor CXCR4 (71, 72). Cxcl-12 acts synergistically with VEGF-A, to regulate tumor vasculature under hypoxic conditions (73). It has been widely demonstrated that Cxcl-12 promotes tumor growth and malignancy, enhances tumor angiogenesis, participates in tumor metastasis, and contributes to immunosuppressive networks within the tumor microenvironment in several types of tumors including breast, prostate, ovarian, colon and non-small cell lung cancer (48, 49, 74). Previous studies have identified different sources of Cxcl-12 in the tumor microenvironment: activated stromal fibroblasts (75), monocytes (76), endothelial cells (77) and even primary tumor cells (78, 79). Interestingly, CXCL-12 has been reported to be expressed by monocytes in an autocrine/paracrine loop to promote differentiation into pro-angiogenic and immunosuppressive macrophages (76). We show here that MK2 regulates Cxcl-12 expression in tumor-infiltrated macrophages, as both myeloid and whole-body MK2 KO mice show deficient Cxcl-12 expression in colon tumors, and adoptive transfer of WT macrophages into myeloid-MK2 KO mice is sufficient to restore Cxcl-12 expression levels, tumor angiogenesis and tumor progression (**Figures 4C–E**). Our data place, for the first time, the chemokine Cxcl-12 under control of MK2 signaling in tumor-associated macrophages to promote angiogenesis (**Figure 5**). The exact molecular mechanism through which MK2 controls Cxcl-12 RNA expression, however, remains to be determined. MK2 could control Cxcl-12 transcription or mRNA stabilization. In the case of pro-inflammatory cytokines and cell cycle regulators that contain AU-rich elements in their 3’UTR, MK2 is known to enhance their mRNA stability by phosphorylating RNA-binding proteins with AU-rich binding domains such as TTP and hnRNPA0 (16, 80, 81). However, we have been unable to discern a clear AU-rich element in the Cxcl-12 3’-UTR. Alternatively, MK2 could regulate one or more transcription factors, or regulate a specific microRNA or non-coding RNA that subsequently targets Cxcl-12 transcripts, as shown previously for MK2 upregulation of miR-34c, which in turn, represses c-Myc (82). However, microRNAs and non-coding RNAs were not included in our RNA expression analysis. In future studies we hope to address these possibilities.

Our results implicating a critical role for MK2 activity in macrophages for inflammatory colon tumor angiogenesis, however, contrast with those of Henriques et al, who concluded that mesenchymal activity of the MK2/Hsp27 axis exclusively contributes to tumor neo-angiogenesis in a APCmin intestinal tumorigenesis model. This disparity may result from differences between the two models (genetic versus inflammation driven) and the target tissue, since APCmin mice develop mostly small intestinal tumors whereas AOM/DSS tumors mostly arise in the distal colon (42). While our experiments cannot completely rule out some contribution of the mesenchymal MK2/Hsp27 axis to tumor angiogenesis as proposed by Henriques et al., the data presented here strongly implicate the importance of macrophages in regulating angiogenesis in the AOM/DSS colon cancer model, which is consistent with a widely recognized role of macrophages in regulating tumor angiogenesis is a variety of cancer types (83, 84).

Macrophages are the most abundant cellular component of the tumor microenvironment (85, 86) and there is strong evidence that these TAMs influence tumor progression at multiple levels. We show here that immune-mediated depletion of macrophages is sufficient to disrupt tumor angiogenesis and tumor progression (**Figures 3B, C**), highlighting the relevance of this cellular compartment for colon cancer progression in this inflammatory model of colon cancer. Our results support growing evidence targeting TAMs maybe a viable strategy for cancer treatment (83) and is consistent with related findings of a reduction in tumor numbers and size following chlodronate-mediated depletion of macrophages in the AOM/DSS model, which was also associated with alterations in the microbiome (87).

This work is of potential importance for cancer therapeutics, as small molecule inhibitors of MK2 are currently in active development (17, 88, 89). Our data indicates that MK2 inhibition could be a promising strategy to block the pro-angiogenic function of tumor-associated macrophages, and therefore may represent an additional weapon with which to combat cancer progression in the near future.

## Data Availability Statement

The original contributions presented in the study are publicly available. This data can be found here: https://www.ncbi.nlm.nih.gov/geo/query/acc.cgi?acc=GSE163716.

## Ethics Statement

The animal study was reviewed and approved by MIT Committee on Animal Care (CAC).

## Author Contributions

LS-L and MY conceived the project, designed the experiments, and analyzed data. LS-L, GS, SR, and SM conducted experiments. YK and JP processed and analyzed RNA sequencing data. KH and MY supervised the project and acquired funding. LS-L, YK, and MY wrote the manuscript. All authors revised the manuscript. All authors contributed to the article and approved the submitted version.

## Funding

LS-L is a recipient of the Research Fellowship award from Crohn’s and Colitis Foundation of America No. 346496. This project was funded by NIH grants R01-ES015339, R01-CA226898 and R35-ES028374, Center grants P30-CA14051 and P30-ES002109, Starr Cancer Consortium Award I9-A9-07, the Charles and Marjorie Holloway Foundation, and the MIT Center for Precision Cancer Medicine.

## Conflict of Interest

The authors declare that the research was conducted in the absence of any commercial or financial relationships that could be construed as a potential conflict of interest.

## Supplementary Material

Supplementary Figure 1 Macrophage depletion in peritoneal cavity and colon following anti-CSFR1 antibody administration. Mice were dosed with 1mg of either control IgG **(A)** or anti-CSFR1 **(B)** antibodies intraperitoneally. Seven days later both peritoneal cavity lavages and colons were harvested and processed for FACS analysis. Macrophages were identified as CD45+, CD11b+, F4/80+ cells. Percentages are relative to total CD45 positive live cells and data is representative of 2 biological replicates.

Supplementary Figure 2 Macrophage recruitment and tumor angiogenesis in Myeloid-specific MK2 KO mice. Representative pictures of **(A)** global macrophage marker F4/80, **(B)** M2 marker Arginase-1 and **(C)** blood vessels marker CD31 detected by immunohistochemistry in colon tumors from LysM-FLFL and LysM-MK2-KO mice. As reported previously, myeloid-specific inhibition of MK2 activity did not significantly affect the total number of macrophages recruited to tumors (F4/80 positive pixel/mm^2^: LysM-FL: 2435 +/- 938.5 vs LysM-KO: 815.3+/-242.6), but significantly reduced M2-macrophages recruitment (ARG-1 positive cells/mm^2^, LysM-FLFL: 362.3 +/- 64.70, LysM-KO: 177.7 +/- 40.26) and tumor angiogenesis (CD31 positive pixels/mm^2^, LysM-FL: 88879+/-8175 vs. LysM-KO: 66380+/-5580). This blinded quantification and statistics can be found in our previous publication (25).
